# The mere exposure effect depends on an odor’s initial pleasantness

**DOI:** 10.3389/fpsyg.2015.00920

**Published:** 2015-07-03

**Authors:** Sylvain Delplanque, Géraldine Coppin, Laurène Bloesch, Isabelle Cayeux, David Sander

**Affiliations:** ^1^Swiss Center for Affective Sciences, University of Geneva, Geneva, Switzerland; ^2^E3 Lab, University of Geneva, Geneva, Switzerland; ^3^Firmenich SA, Geneva, Switzerland

**Keywords:** mere exposure, olfaction, pleasantness, familiarity, preference

## Abstract

The mere exposure phenomenon refers to improvement of one’s attitude toward an a priori neutral stimulus after its repeated exposure. The extent to which such a phenomenon influences evaluation of *a priori emotional* stimuli remains under-investigated. Here we investigated this question by presenting participants with different odors varying in *a priori* pleasantness during different sessions spaced over time. Participants were requested to report each odor’s pleasantness, intensity, and familiarity. As expected, participants became more familiar with all stimuli after the repetition procedure. However, while neutral and mildly pleasant odors showed an increase in pleasantness ratings, unpleasant and very pleasant odors remained unaffected. Correlational analyses revealed an inverse U-shape between the magnitude of the mere exposure effect and the initial pleasantness of the odor. Consequently, the initial pleasantness of the stimuli appears to modulate the impact of repeated exposures on an individual’s attitude. These data underline the limits of mere exposure effect and are discussed in light of the biological relevance of odors for individual survival.

## Introduction

More than 40 years ago, Zajonc (1968) presented his seminal work showing that “*repeated, unreinforced exposures produce an enhancement in affect toward a stimulus*” (p. 1). Since then, this *mere exposure effect* has become one of the most inspiring and studied phenomena in psychology (Bornstein, 1989; Moreland and Topolinski, 2010). In the classical paradigm used to investigate the mere exposure effect, participants are presented with a series of stimuli at different exposure frequencies within a limited time window. At a certain point, they are requested to rate their preference toward the stimuli. Experimental manipulations such as stimulus type, duration, presentation frequency, and type of ratings, as well as personality and individual variables, have been extensively studied (see Bornstein, 1989, for a review). A robust phenomenon, the mere exposure effect has been replicated in hundreds of experiments using visual, auditory (Bornstein, 1989), olfactory (e.g., Prescott et al., 2008), and recently, haptic stimuli (Jakesch and Carbon, 2012). This effect has been found even when stimuli are presented subliminally (e.g., Bornstein and D’Agostino, 1992). Hence, the mere exposure effect seems to impact any situation during which one is confronted with stimulus repetitions. It is consequently thought to constitute a key element in preference acquisition (e.g., Balogh and Porter, 1986; Schaal et al., 2000).

The vast majority of data on the mere exposure effect have been collected on meaningless *neutral* visual stimuli. In Zajonc’s (1968) princeps study, for example, the subjects did not usually have “*a prior preference for the stimulus exposed*” (p. 23). The extent to which exposure could influence preferences or hedonic ratings of *a priori emotional* stimuli has rarely been investigated. This is surprising, given that encountering neutral^1^ stimuli could constitute the exception, rather than the norm, in daily life. Studies examining the mere exposure effect in relation to *a priori* valenced stimuli are scarce: Although they all indicate that the initial pleasantness of a stimulus is an important variable to consider, the impact of the mere exposure effect ranges from canceling out preferences to strengthening them. For instance, Schellenberg et al. (2008) did not find any differential exposure influence on pleasantness evaluation of happy and sad musical pieces. Grush (1976) suggested that *a priori* pleasant, meaningful words became more pleasant after repeated exposures whereas *a priori* unpleasant words became more unpleasant. Evidence also suggests that exposure can improve hedonic evaluations of initially disliked harmless and caged living snakes (Litvak, 1969) and can reduce the dislike of angry faces (Young and Claypool, 2010). Using a modified prisoner’s dilemma, Swap (1977) reported observing more important exposure effects (i.e., increases in interpersonal reported attraction) for rewarding partners than for punishing partners.

In the olfactory domain and with correlational approaches, several authors have described an increase in the reported pleasantness of odors with their familiarity (e.g., Engen and Ross, 1973; Lawless and Cain, 1975; Ayabe-Kanamura et al., 1998; Distel et al., 1999; Royet et al., 1999; Bensafi et al., 2002; Sulmont et al., 2002). However, Delplanque et al. (2008) showed that the correlation between pleasantness and familiarity is much more important for pleasant odors than for unpleasant ones (correlations were *not* significant for malodors). Similar results were since obtained with various set of odorants across the world (Ferdenzi et al., 2013). These results suggest that malodors are resistant to pleasantness increases that could be expected from exposure. The authors underlined the adaptive advantage of unpleasant odor processing in allowing individuals to avoid, as much as possible, the influence of exposure to the odorant (i.e., increasing familiarity) in order to maintain negative attitudes toward a potentially dangerous stimulation.

Investigating the mere exposure effect with *a priori* valenced stimuli may appear to be challenging since many studies used meaningless stimuli, e.g., geometric abstract shapes that are not valenced. In visual or auditory modalities, valenced stimuli are likely to be explicitly meaningful, as they are subjected to many regulations and high-level interpretations that could influence the mere exposure effect. In a classic review of mere exposure studies, Bornstein (1989, p. 275) highlighted “*that stimulus recognition may actually inhibit the exposure effect.*” Olfactory stimuli are putative perfect candidates in that sense, since their pleasantness is thought to be the major representation of human odorant perception (Yeshurun and Sobel, 2010) and humans do not perform well in explicit odor recognition (Issanchou et al., 2002; Stevenson, 2009).

Not only are studies investigating the mere exposure effect in relation to the *a priori* valence of stimuli scarce, but they are mainly correlational, which considerably narrows their explanatory power. They cannot demonstrate that a change in familiarity, due to exposure, *causes* a change in pleasantness. Moreover, they cannot prove that those putative changes are different along the pleasantness continuum.

In an attempt to fill this gap, the aim of the present experiment was to investigate the impact of the initial pleasantness of stimulus on the mere exposure effect by directly manipulating exposure to unpleasant, neutral, and pleasant olfactory stimulations. More precisely, we implemented a familiarization procedure for six odorants that varied in pleasantness. To avoid any confound between a mere exposure effect and habituation or desensitization effects (that are known to occur rapidly in olfaction; Cain and Johnson, 1978; Comeno-Muniz and Cain, 1995), or affective habituation (Ferdenzi et al., 2014), we did not present the odorant intensively during one session. Rather, we organized six judgment sessions separated by at least 1 day. During one session, odorants were randomly presented and participants had to rate the pleasantness, the familiarity, and the intensity of each of them. Participants’ ability to recognize and label odors could not only influence their familiarity and pleasantness evaluations (Seo et al., 2008), but also the mere exposure effect itself (Bornstein, 1989). In order to assess such potential confounds linked to odors recognition, we performed a free and cued odor recognition task at the end of the familiarization procedure. In sum, if unpleasant odors are more resistant to mere exposure effect, as a previous correlational study suggests (Delplanque et al., 2008; Ferdenzi et al., 2013), we expected that changes in pleasantness ratings after repeated exposures would be less important for initially unpleasant odors than for initially neutral or pleasant ones.

## Materials and Methods

### Participants

Forty participants (21.72 ± 2.94 years, 10 males) took part in this experiment. They were paid 20 Swiss francs for their participation. Before starting the experiment, participants completed a consent form. They all self-reported a normal sense of smell. Participants gave written informed consent, and the study was approved by the ethical committees of the Psychology Department of the University of Geneva.

### Stimuli

Six odorants provided by Firmenich, S.A. were selected [isovaleric acid (cheese), skunk (feces), leather, lilac, shampoo fragrance, and strawberry] on the basis of pleasantness ratings obtained in preceding studies (Delplanque et al., 2008; Chrea et al., 2009). Solutions (6 ml) of these odorants were injected into the absorbent core of cylindrical felt-tip pens (14 cm long, inner diameter 1.3 cm), using the same concentrations as in preceding studies (Delplanque et al., 2008; Chrea et al., 2009). Moreover, a small sample of Firmenich employees checked the concentrations in the pens to ensure that the odors were subjectively judged as (1) well perceived without being too strong and (2) without any notable difference in perceived intensity across all odorants. The use of this highly practical system provided by Burghart (Germany) prevents contamination by the environment. An additional pen without any odorant (blank pen) was added to the selection. Each odorant was coded by a random three-digit code and these codes were changed during the experiment to avoid recall across different sessions.

### Procedure

Participants completed six judgment sessions, each separated by at least 1 day (median = 3, minimum = 1, maximum = 19). Data collection lasted 5 weeks. During each session, participants smelled the seven odor pens in random order. The interval between two odorants varied from 30 to 45 s to avoid sensory adaptation. Before testing, participants were instructed on how to smell the odorants in order to minimize the intra- and inter-participant breathing pattern variability. The instructions were as follows: when the participants saw the three-digit code on the screen, they had to (1) take the corresponding pen from the display shelf; (2) uncap the pen and breathe evenly for only one sniff with the odorant pen near the nose (about 1 cm below both nostrils); (3) cap the pen, put it back on the display shelf; and (4) use the three scales (described in detail in the next section) and wait for the signal to proceed to the next trial.

### Scales and Measures

In each session, participants had to complete a computer-based questionnaire. For each odorant, they were asked to judge the pleasantness, from “very unpleasant” (left side of the scale = 0) to “neutral” (middle of the scale = 300) to “very pleasant” (right side of the scale = 600); the familiarity from “not familiar at all” (left = 0) to “medium” (middle = 300) to “very familiar” (right = 600); and the subjective intensity from “not perceived” (left = 0) to “medium” (middle = 300) to “very strong” (right = 600) by placing a cursor on the continuous scale with the mouse. Participants were also informed that they could use all of the intermediate positions. At the beginning of each session, they were also asked to rate the subjective level of their hunger on a four-point scale (not at all, slightly, mildly, and strongly). At the end of the last session, they performed a free identification task during which they had to guess each odorant’s name. A response was considered as correct if the participant gave the exact name of the odorant source or its synonyms (e.g., manure for feces, soap for shampoo) or the relative category (e.g., flower for lilac, cosmetic for shampoo). This was followed by a cued recognition task (similar to the Sniffin’ Sticks recognition test) during which they had to find each odorant’s name included in a series of three other wrong alternatives.^2^

## Results

### Initial Ratings

**FIGURE 1:**
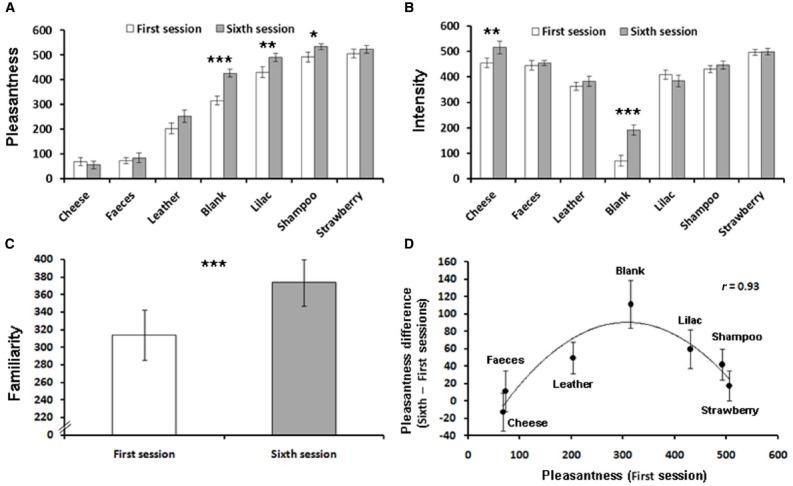
**(A)** Mean pleasantness ratings for the first and sixth sessions for each odor. **(B)** Mean intensity ratings for the first and sixth sessions for each odor. **(C)** Mean odor familiarity ratings for the first and sixth sessions. **(D)** Mean pleasantness difference (sixth—first session) for each odor as a function of its initial pleasantness (first session).*p < 0.05, **p < 0.01, ***p < 0.001; error bars represent standard error of the mean; minimum/maximum for all scales = 0/600.

At the beginning of the experiment, before any experimental exposure procedure, participants’ agreement about the pleasantness of odors was high (Cronbach’s alpha = 0.990; average inter-rater correlation = 0.830). The participants clearly differentiated the pleasantness of the odors [Greenhouse–Geisser corrected (G-G) repeated measures analysis of variance (ANOVA); *F*(6,234) = 107.9, *p* < 0.001, η^2^ = 0.73]. Further analyses (Tukey HSD *post hoc* comparisons) revealed that all the odors were significantly different except the pair feces and cheese on the one hand and the pairs lilac/shampoo and shampoo/strawberry on the other hand (see Figure 1A, first session). Thus, the set of odors was composed of two unpleasant stimuli (feces and cheese), two neutral stimuli (leather and blank pen), and three pleasant stimuli (lilac, shampoo fragrance, and strawberry).

Familiarity ratings were also different across odors [*F*(6,234) = 21.8, *p* < 0.001, η^2^ = 0.35], and subsequent *post hoc* analyses revealed two groups of odors. A group of similarly highly familiar odors, composed of lilac, strawberry, and shampoo, was distinguished from another group of less familiar but similar odors, composed of cheese, feces, leather, and the blank pen.

Odor intensities were also evaluated differentially [*F*(6,234) = 74.86, *p* < 0.001, η^2^ = 0.65; see Figure 1B, first session]. The blank pen was significantly evaluated as less intense than all the other odors (*post hoc* Tukey HSD), as was the leather odor, except in comparison with lilac. Finally, strawberry was evaluated as significantly more intense than lilac.

To examine whether our odor sample was characterized by the classical positive correlation between familiarity and pleasantness, we examined the relationship between the subjective variables (Pleasantness, Familiarity, and Intensity) assessed during the first session. There was a linear and positive correlation between the pleasantness and the familiarity of the odors (Pearson *r* = 0.86, *p* < 0.05). However, the quadratic regression was also significant and the regression coefficient was more important [*r* = 0.93, *F*(2,4) = 14.7, *p* < 0.05], highlighting the weakness of the pleasantness–familiarity relationship for unpleasant odors, the correlation being reinforced as the pleasantness increased. We did not find any other significant linear or quadratic relations between the subjective measures.

### Influence of Exposure on Familiarity Evaluation

To test the effectiveness of our paradigm in inducing the expected increase in evaluation of the familiarity of odors after exposure, we conducted a G-G repeated measures ANOVA with Odor (six levels) and Session (two levels) on familiarity ratings obtained in the first and sixth sessions. The main effect of Session was significant [*F*(1,39) = 7.75, *p* < 0.001, η^2^ = 0.24], showing an increase in familiarity ratings between the two sessions (see Figure 1C). Neither the main effect of Odor nor the interaction reached significance. Thus, the procedure induced familiarization for all odors, i.e., an increase in familiarity ratings between the first and the last sessions.

### Influence of Exposure on Pleasantness Evaluation

Participants’ agreement about odor pleasantness was still high after repeated exposures to odors (Cronbach’s alpha = 0.993; average inter-rater correlation = 0.878). A G-G corrected repeated measures ANOVA with Odor (six levels) and Session (two levels) was performed on the pleasantness ratings obtained in the first and sixth sessions. A significant Odor × Session interaction was observed [*F*(6,234) = 3.6, *p* < 0.001, η^2^ = 0.08]. ANOVAs performed for each odor revealed a marginally significant increase in pleasantness for leather [*F*(1,39) = 3.22, *p* = 0.08] and significant increases in pleasantness for the blank pen, lilac, and shampoo odors [*F*_*s*_(1,39) = 25.45, 7.2, 5.47; *p*_*s*_ < 0.001, 0.01, 0.05; η^2^_*s*_ = 0.39, 0.15, 0.12, respectively; see Figure 1A]. Thus, pleasantness representation was affected by repeated exposures, a significant increase in pleasantness with familiarization being observed only for neutral/mildly pleasant odors, but not for unpleasant or very pleasant odors.

Regression analyses were also conducted on the difference of pleasantness ratings between the sixth and the first sessions related to the pleasantness ratings of the first session. We observed a strong and significant quadratic regression [*r* = 0.93, *F*(2,4) = 12.6, *p* < 0.05; see Figure 1D] that remained significant when the blank pen was removed [*r* = 0.93, *F*(2,3) = 10.59, *p* < 0.05], revealing an inverse U-shape relation between pleasantness increase caused by exposure and initial pleasantness of the odor.

### Influence of Exposure on Intensity Evaluation

The G-G corrected repeated measures ANOVA with Odor (six levels) and Session (two levels) performed on the intensity ratings obtained in the first and sixth sessions revealed a significant Odor × Session interaction [*F*(6,234) = 6.98, *p* < 0.001, η^2^ = 0.13]. ANOVAs performed for each odor revealed significant increases in intensity for the blank pen and cheese odor [*F*_*s*_(1,39) = 19, 9.45; *p*_*s*_ < 0.001, 0.01; η^2^_*s*_ = 0.33, 0.19, respectively; see Figure 1B]. The linear correlation conducted on the difference of pleasantness and the intensity ratings between the sixth and the first sessions was not significant. This result renders the influence of intensity changes on the observed pleasantness changes due to exposure very unlikely.

### Identification Scores and Hunger Level

The percentage of correct identification obtained during the free identification task was globally low (38%) but differed across odorants [Cochran Q Test, *Q*(5) = 44.18, *p* < 0.001], increasing from cheese (12.5%) to feces and lilac (27.5%), leather (32.5%), shampoo (60%), and strawberry (67.5%). Percentages obtained in the cued recognition task were high, varying from 80 to 97% (mean = 88%) of correct responses, but were not significantly different across odorants. The mean reported hunger state was low (0.83), varying from 0.65 to 1.15, and did not significantly differ across sessions.

## Discussion

In this study, we aimed to investigate the impact of the initial pleasantness of olfactory stimuli on the mere exposure effect. More precisely, odorants varying in pleasantness were presented once during six judgment sessions separated by at least 1 day to avoid any confound between a mere exposure effect and habituation or desensitization effects. This exposure procedure induced an increase in familiarity for all odors, confirming its efficiency. As expected, change in familiarity, due to exposure, caused changes in pleasantness. In particular, neutral and mildly pleasant odors were evaluated as more pleasant after the exposures than during the first session. However, these changes in pleasantness were not observed for odors that were initially unpleasant or very pleasant. The observed pattern of results is unlikely to be due to peripheral habituation since each odor was smelled only once during a particular session, and each session was separated from another by at least 1 day. In the same vein, it is unlikely that affective habituation played a role here, as intensive exposure to initially pleasant odors has been shown to reduce their pleasantness, whereas intensive exposure to initially unpleasant odors increases their pleasantness (Cain and Johnson, 1978), a pattern inconsistent with the one obtained in this study. The present data suggest that mere exposure effect is predominantly observed when initial odor evaluations are not strongly polarized on the pleasantness continuum.

As hypothesized, malodor evaluations were more resistant to the influence of repeated exposures. This result is consistent with the absence of a correlation between pleasantness and familiarity for malodors observed in correlational studies (Delplanque et al., 2008). From a functional perspective, it seems adaptive for malodor processing to allow individuals to avoid, as much as possible, the influence of exposure in order to maintain negative attitudes toward a potentially dangerous stimulation. By contrast, pleasantness evaluation of *a priori* neutral/mildly pleasant odors was affected by repeated exposures, which led to an enhancement in affect toward them. This last result constitutes the typical mere exposure effect as first described by Zajonc (1968). The gain in pleasantness due to exposures could favor approach behaviors to explore and gain information from potentially beneficial situations. The most important influence was observed for the pure neutral stimulus, i.e., the pen without odor. It is unlikely that this point has biased the whole pattern of results, since the quadratic regression conducted without this stimulus was still significant, showing that the inverse U-shape we observed was not due to this particular stimulus. This example likely better reflects that the mere exposure effect is optimally obtained for neutral stimuli.

The unexpected result of this experiment was that the most *a priori* pleasant odor’s hedonic evaluation was not affected by repeated exposures. Even though this result was observed only for this most *a priori* pleasant odor (i.e., strawberry aroma), the regression analysis showed that the gain in pleasantness due to exposures weakened as pleasantness increased. This result means that less enhancement of preference occurs with exposures to an *a priori* pleasant stimulus than with an *a priori* neutral stimulus. One can wonder whether this result could be due to a rating bias, the initial pleasantness being already too high and reaching a ceiling that prevented further increases in pleasantness ratings with repeated exposures. However, the remaining space available on the scale was, on average, very close (94.8/600) to the largest pleasantness changes due to exposures (111.1/600) that was obtained for the blank pen. There was consequently potential space for increased evaluation. A more plausible explanation would be that pleasant odors are spontaneously better identified, this recognition decreasing the magnitude of the mere exposure effect as is thought to be the case with other modalities (Bornstein, 1989). A supplementary correlational analysis performed on our data revealed a significant positive linear increase in recognition success with pleasantness (Pearson *r* = 0.86, *p* < 0.05). Alternatively, when the pleasantness is initially very meaningful, there is less room for further learning and change, as the consequences of being exposed to the pleasant stimuli are well known and need no further adaptation. Thus the mechanism of increasing pleasantness to favor an approach is no longer beneficial. This interpretation could explain why there is a positive correlation between familiarity and pleasantness for *a priori* pleasant odors, as observed in correlational studies, together with the fact that pleasantness will not be further reinforced for most pleasant odors with repeated exposure, as demonstrated in our study.

The typical proposed mechanism underlying the mere exposure effect is that previous exposures to a stimulus enhance its perceptual fluency, making it more prototypical and familiar. Greater fluency then automatically generates a more positive affect that modifies pleasantness evaluation. This fluency explanation has received much experimental support in other sensory modalities (see Moreland and Topolinski, 2010, for a discussion on this topic). Sulmont et al. (2002) brought forward elements in favor of this idea in the olfactory domain by reporting that the more familiar and pleasant the odors, the simpler they are perceived by participants, whereas the number of perceived notes remained relatively independent of familiarity, suggesting that simplicity is not related to physical complexity. In this framework, our results suggest that only odors that are not *a priori* too polarized on the pleasantness continuum benefit from this fluency effect. One could speculate that this fluency gain would be inhibited for malodors, whereas fluency would reach a plateau and not be further enhanced when odors are highly pleasant.

The study of the underlying processes of the mere exposure effect has recently benefited from a new line of research based on the incorporation of the embodiment concepts in the fluency hypothesis (e.g., Moreland and Topolinski, 2010). According to this embodied fluency hypothesis, not only would the perceptual representation of a stimulus become more fluent due to repeated exposures, but so too would the stimulus-related sensorimotor simulations (Beilock and Holt, 2007; Topolinski and Strack, 2009, 2010), since embodiment theories postulate that the stimuli representations include the sensorimotor responses associated with those stimuli (e.g., Niedenthal et al., 2005, 2009; Semin and Smith, 2008). Sniffing patterns reflecting odor pleasantness (Bensafi et al., 2003), a new line of research could investigate whether changes in the pleasantness of odors with repeated exposures are related to a specific breathing pattern (e.g., Ferdenzi et al., 2014).

In sum, this study demonstrates that mere exposure effect optimally hold for neutral and mildly pleasant olfactory stimuli and are dramatically reduced for either unpleasant or pleasant stimuli. Although this result remains to be confirmed for other sensory modalities, it suggests that mere exposure does not similarly impact all situations during which one is confronted with stimulus repetitions: Initially unbearable or exquisite events will continue to be so.

### Conflict of Interest Statement

The authors declare that the research was conducted in the absence of any commercial or financial relationships that could be construed as a potential conflict of interest.

## Footnotes

1 In the literature on classical conditioning, a neutral stimulus is one without intrinsic motivational properties that has never been conditioned with a motivationally or emotionally relevant stimulus (see Rescorla, 1967; Balleine and Killcross, 2006; Esber and Haselgrove, 2011, for reviews).

2 The different series of terms were (correct name in italics): Orange/Pineapple/*Strawberry*/Cassis, *Leather*/Smoke/Grass/Glue, Ham/*Cheese*/Bread/Fish, Pear/Pineapple/Prune/*Lilac*, Ammonia/Tobacco/*Feces*/Turpentine, and Chamomile/*Shampoo*/Grapefruit/Apple.
